# 3D Printable Soft Sensory Fiber Networks for Robust and Complex Tactile Sensing

**DOI:** 10.3390/mi13091540

**Published:** 2022-09-17

**Authors:** David Hardman, Thomas George Thuruthel, Antonia Georgopoulou, Frank Clemens, Fumiya Iida

**Affiliations:** 1 Bio-Inspired Robotics Laboratory, Department of Engineering, University of Cambridge, Cambridge CB2 1PZ, UK; 2 Department of Functional Materials, Empa-Swiss Federal Laboratories for Materials Science and Technology, Überlandstrasse 129, 8600 Duebendorf, Switzerland; 3 Brubotics, Vrije Universiteit Brussel (VUB), Pleinlaan 2, B-1050 Brussels, Belgium

**Keywords:** soft robotic sensors, 3D printing, machine learning

## Abstract

The human tactile system is composed of multi-functional mechanoreceptors distributed in an optimized manner. Having the ability to design and optimize multi-modal soft sensory systems can further enhance the capabilities of current soft robotic systems. This work presents a complete framework for the fabrication of soft sensory fiber networks for contact localization, using pellet-based 3D printing of piezoresistive elastomers to manufacture flexible sensory networks with precise and repeatable performances. Given a desirable soft sensor property, our methodology can design and fabricate optimized sensor morphologies without human intervention. Extensive simulation and experimental studies are performed on two printed networks, comparing a baseline network to one optimized via an existing information theory based approach. Machine learning is used for contact localization based on the sensor responses. The sensor responses match simulations with tunable performances and good localization accuracy, even in the presence of damage and nonlinear material properties. The potential of the networks to function as capacitive sensors is also demonstrated.

## 1. Introduction

Soft robotic sensing technologies have shown incredible developments in the last decade [1]. Their applications are wide and diverse, including the fields of soft robotics, wearable devices and human–machine interfaces. Although these novel technologies are promising, their usage has been limited by difficulties in fabrication, design and modeling: issues that are interdependent [2,3]. This work combines novel developments in 3D printing, flexible materials, and information theory-based design and modeling approaches to develop more accurate and robust sensory skins.

Functional materials used in the design of soft strain sensors include liquid conductors [4,5,6], nanocomposites [7,8,9], and optic fibers [10]. Fabrication and placement of these sensors typically involve direct moulding, injection moulding and/or manual placement. These processes introduce variability among the sensors. Additionally, these sensors suffer from highly nonlinear time-dependent effects. Depending on the strain responsive mechanism, this can be because of damage, rearrangement of conductive particles [11], geometric effects induced by the surrounding viscoelastic matrix, or delamination [12]. Precise and repeatable manufacturing techniques can introduce methods for reducing these nonlinear effects and studying their cause [13,14]. At present, 3D printing sensory structures is one of the ways to improve sensor properties [15,16,17,18,19,20,21,22]; it not only allows us to fabricate sensors in a consistent and repeatable manner, but also allows us to develop complex sensor morphologies that are designed through optimization.

The role of sensor morphology and its application for the processing and structuring of sensory cues is a commonly observed phenomenon in nature and often applied in robotic sensors [23]. Two examples in nature are the facial whiskers of rodents [24] and the sensilla morphology of the crayfish antennular flagellum [25]. Similar concepts have been applied to soft robotic sensors: Culha et al. used finite element models to find the optimal sensor morphologies for detecting kinematic parameters [26]. Optimization techniques can be applied to select sensor combinations & layouts based on a user-defined set of characteristic deformation modes [27,28]. By simulating the behaviors of their proprioceptive sensors, Tapia et al. determine the placement of silicone tubes in an injection-molded bar, which are manually routed and cast in place [29]. A previous work by the authors used genetic algorithms to optimize soft strain sensor morphologies which perform better in terms of robustness and accuracy [30], important properties for robots deployed in hazardous or remote environments [31]. However, due to poor manual fabrication techniques, these optimized sensor networks had drastic sim-to-real differences. Machine learning techniques present a promising route to accounting for discrepancies in sensor response: using 132,000 samples and a 6-layer neural network, Sun et al. achieved superresolution in their manually fabricated taxel-based soft skins [32].

**Figure 1:**
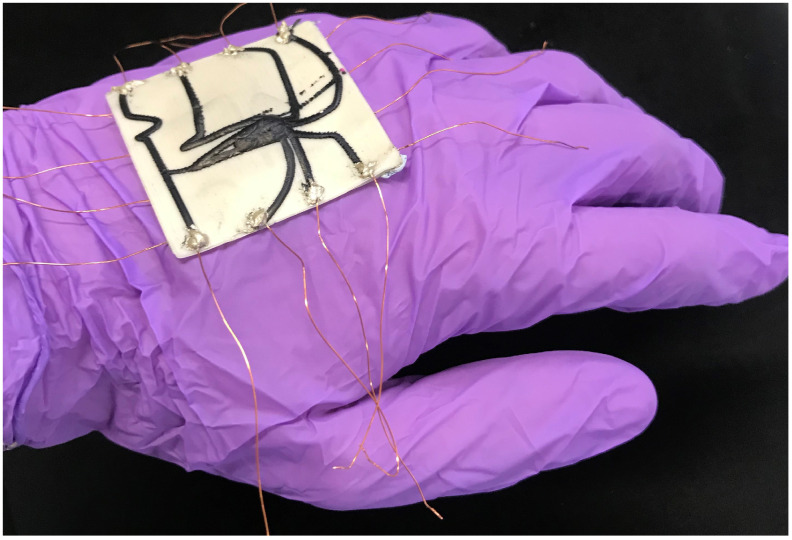
A 3D-printed soft sensory fiber network functioning as a flexible skin.

This work demonstrates how 3D printing technology can improve the predictability of soft sensors and, hence, unlock model-based sensor optimization techniques (Figure 1). We first introduce elastomeric materials and fabrication techniques, using pellet-based 3D printing to implement the optimized sensor morphologies. To test this approach, we print and compare two different sensor networks designed by Thuruthel et al. [30], each consisting of eight piezoresistive sensorized channels on a dielectric substrate. We characterize their responses as they are pressed with a robotic end effector, before using this setup to train a neural network with real-world data, comparing its output to previous analytic simulations. Our results demonstrate how predictable performances that match simulation models can be obtained through 3D printing technology which can further help study the performance of these sensory skins under different conditions. We also investigate the applicability of our 3D-printed sensory skins for capacitive sensing, in order to measure applied forces and provide additional redundancy to the network.

## 2. Fabrication

**Figure 2:**
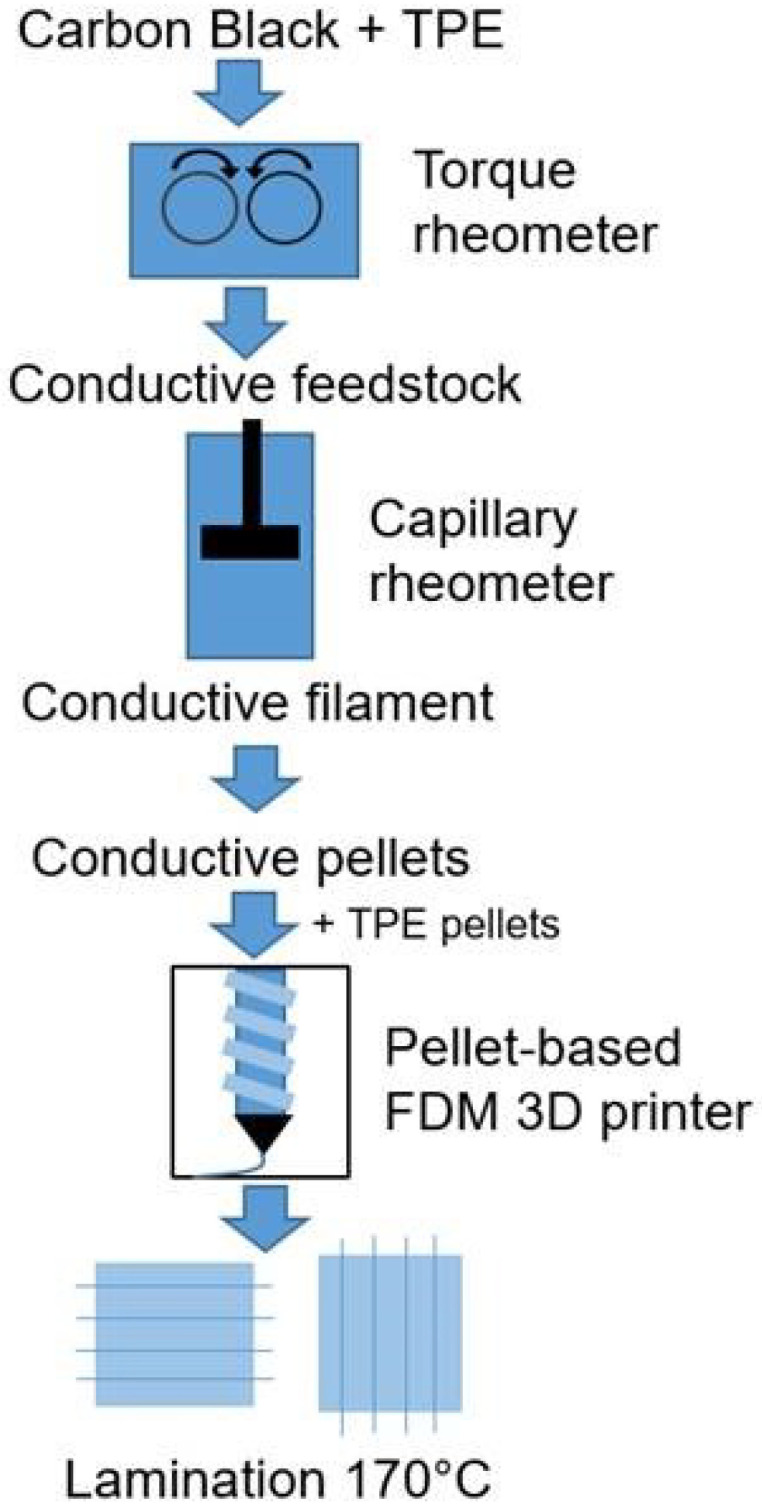
Fabrication steps of the sensor fiber networks.

**Figure 3:**
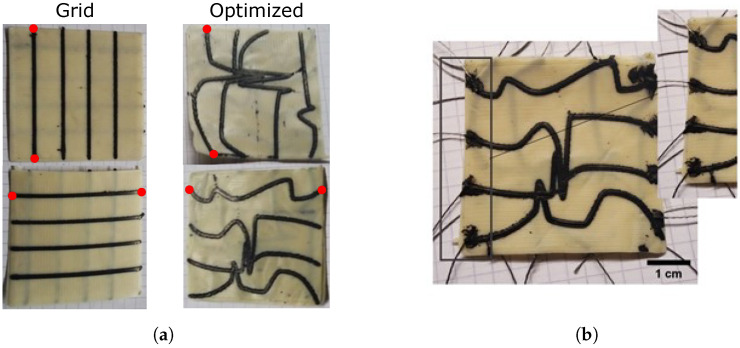
(**a**) The 3D-printed sensing fiber networks before lamination, fabricated with pellet-based FDM for the grid and optimized morphologies. Red dots mark the channels which are later assumed to be broken during damage characterization. (**b**) Connection of the conductive yarn with conductive paste on the edge of the 3D-printed optimized sensing fiber network.

The complex sensory skins which we present are fabricated entirely using 3D printing, combining two soft and flexible materials—a piezoresistive sensing material and an elastomeric substrate—into a composite network. A Voladora NX+ pellet printer (International Technology 3D Printers, S.L., Spain) is used to create the composite material [33], producing two-layer sensors (Figure 2): the piezoresistive material is used to print a specific morphology of sensor network onto a 45 × 45 × 0.4 mm square of the substrate. To create double-sided networks, two such prints are laminated together by placing them in the oven for 30 min at 170 °C. In this work, we demonstrate the advantages in customization offered by this method by fabricating and comparing two networks. The first is a simple 4 × 4 uniform grid, whilst the second uses a morphology that has been optimized by Thuruthel et al. for robustness using genetic algorithms (Section 5.1). The two printed morphologies are shown in Figure 3. Throughout the characterization process, the responses of these skins are compared with the idealized simulated responses obtained during the optimization process, and we find the printed networks to much better match the simulated behaviors when compared to manually fabricated alternatives.

**Figure 4:**
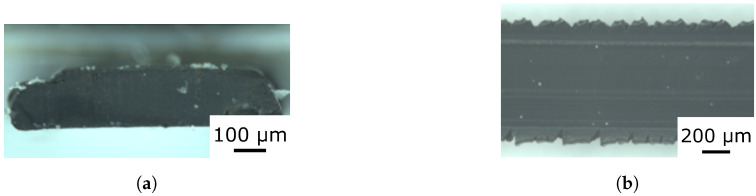
Cross-section and top view of the printed sensory threads creating the 3D sensing fiber network. (**a**) Cross-Section; (**b**) Top View.

Using additive manufacturing, the sensor networks can be produced within 20 min, which is significantly shorter than the time required for casting and curing silicone rubber elastomer sensor networks as reported by Thuruthel et al. [30]. For the network optimized for robustness (details given in Section 5.1), using sensor fibers instead of printed threads is very time consuming and difficult to produce with high accuracy, where additive manufacturing is significantly advantageous compared to manual fabrication. Additional problems such as the proper alignment of the oriented sensor network are not an issue with additive manufacturing, as shown in Figure 4, where the cross-section area of the printing lines is determined using a Zeiss Stereo Discovery microscope (Carl Zeiss Microscopy, Jena, Germany). Though printing the fiber network with an orifice below 0.6 mm results in a blocking of the orifice of the nozzle due to agglomerates of the carbon black, Figure 4 demonstrates a width of 0.68 mm and a thickness of 0.18 mm, which enables the fabrication of complex morphologies onto the 45 × 45 mm elastomer skins.

For the piezoresistive sensing material, a styrene-based tri-block co-polymer TF5 ATL (Kraiburg TPE, Waldkraiburg, Germany) with Shore hardness 50A is used. The material has a density of 0.88 g/cm${}^{3}$, 800% elongation at the point of fracture and a tensile strength of 8.5 MPa. In comparison to other Shore hardnesses, the piezoresistive sensors with TF5 ATL gives the best performance [33]. For the sensor, the styrene based tri-block co-polymer is mixed with the carbon black Ensaco 250G (Imerys, Paris, France) with a BET of 65 m${}^{2}$/g by a torque Rheometer HAAKE Polylab Rheomix 600 (Thermofisher, Waltham, MA, USA). A torque rheometer is a high shear mixer with a torque sensor that conveys information for monitoring the mixing process. The compound is then extruded into strands with a diameter of 1.75 mm using a capillary rheometer RH7 (NETZSCH, Selb, Germany). With this device, the applied force and the pressure during extrusion of the strands can be monitored. In order to achieve granulates with a length of 3 mm, the strand is cut manually. The two sensing fiber network structures—the 4 × 4 grid and optimized morphologies—are printed using a pellet-based fused deposition modeling (FDM) method (Figure 3). In both cases, the width of the printed lines is 0.6 mm and the thickness 0.2 mm. To be able to investigate the piezoresistive and capacitive sensing behavior of the network, both structures are printed on a styrene-based tri-block copolymer (Kraiburg TPE, Germany) elastomeric substrate, with shore hardness of 68A. The layer thicknesses are chosen to provide good resistive and capacitive responses whilst remaining robust; the same technology is capable of printing these sensory grids with a total thickness of 0.3 mm (Figure S1).

**Figure 5:**
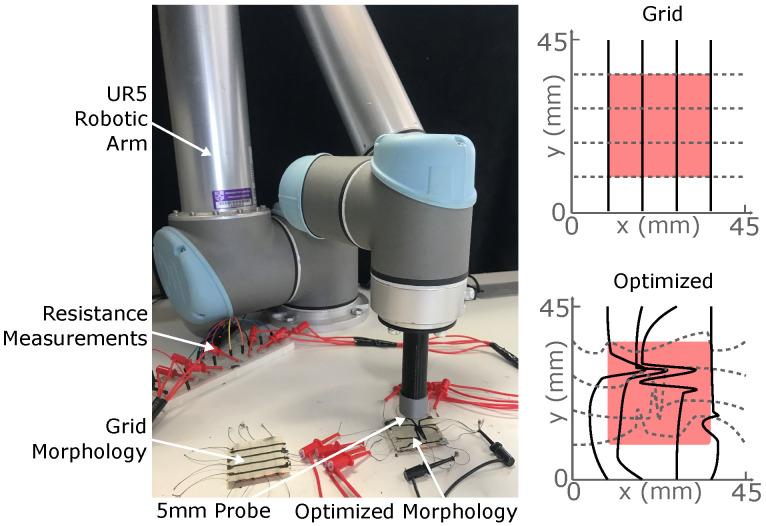
The robotic arm setup for characterizing the two sensor morphologies: grid and optimized. A probe with 5 mm diameter presses 2 mm vertically downwards anywhere within the red shaded area, and the corresponding changes in channel resistances and capacitance are recorded. In the schematics, solid black lines indicate sensor channels that were uppermost during testing, and dashed grey lines those on the underside.

During printing, a temperature of 230 °C is used for the nozzle and 90 °C for the bed. The printing speed is 30 mm/s and the extrusion multiplier is set at the value of 10. Figure 5 shows the two printed fiber networks before the lamination process. After lamination, a conductive yarn (Adafruit, New York, NY, USA) is used to connect the edges of the conductive pathways to the characterization setup (Figure 3b). During the preliminary characterizations, the connective yarn is fixed in place using a conductive paste (Bare Conductive, London, UK), whilst this is replaced with a conductive epoxy (MG Chemicals, Manchester, UK) during later experiments.

## 3. Results

### 3.1. Resistive Sensor Characterization

The characterization of the two sensor networks is undertaken using a robotic arm to provide a series of controlled and precisely located 2 mm-deep presses across the surfaces of the two sensor morphologies: Figure 5. During the presses, we characterize both the resistive and the capacitive responses of the networks, demonstrating their abilities to detect the deformations through both methods. Further details are given in Section 5.2, with additional thorough material analyses performed by Georgopoulou and Clemens [34].

**Figure 6:**
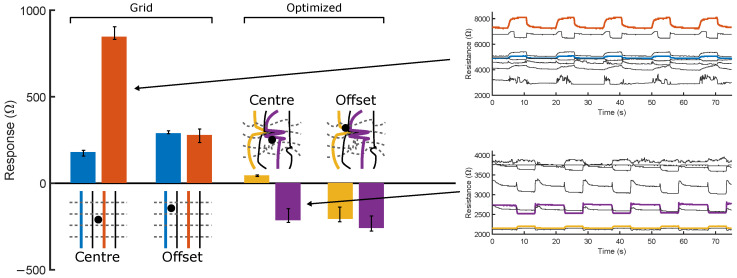
Relative magnitude of the resistive responses at central and offset probe locations. Central and offset positions are probed 10 times for both the grid and the optimized morphologies, with the responses from two of the eight sensor channels (depicted in blue and orange) being plotted. Two characteristic resistive time-series responses to 5 repeated 2 mm-deep probes at the skin’s centre are shown, one from each morphology.

For each morphology, two of the eight sensors are chosen and their resistive response magnitudes to probing at two locations—Centre (22.5, 22.5) and Offset (14.5, 30.5)—are displayed in Figure 6’s bar plot. Each probe is repeated 10 times whilst measuring the maximum deviation from the local baseline resistance, taken to be the average of the measured value directly prior to and after probing, in order to account for any small effects of transient drift. Sensors in the uniform grid demonstrate an increase in resistance when a deformation is applied, matching the assumptions made by Section 5.1’s simulations—that local applied strains will impede the flow of current through the sensor. Similarly, both sensors increase their response magnitudes when the probing location moves towards them—i.e., when moving from the centre to the offset position, the outer (blue) sensor’s magnitude increases, whilst the inner (orange) magnitude falls. Still, variations in print quality (particularly in sensor height and interlayer adhesion) and connections means that all eight sensors do not behave identically—the inner sensor responds much more strongly to a close probe than the outer sensor. As the simulation model is developed purely from the geometric shape of the sensor under deformation, these variabilities should not affect the sim-to-real performance, assuming that the geometric shape of the sensor morphology is the same as the simulation after 3D printing. The time series responses of all eight sensors to five consecutive central probes are plotted in Figure 6: no significant drift or overshoot is detectable in this response. The resistive responses are not only limited to the sensors directly next to the probed location: all eight respond to some extent in these time series plots, likely due to a combination of global deformation and crosstalk between sensors. The magnitudes of all eight responses can be used to train a neural network for localization, which can use their relative values to predict the probed point.

The sign of the optimized morphology’s responses, as well as the magnitudes, can change with probe location: the outer (yellow) sensor is seen to increase in resistance when centrally probed, whilst decreasing in resistance during offset probing. Noting the proximity of the offset location to the tight cluster of sensors, and that the width of the channels in the printed samples lead to contact between adjacent sensors in this area, we hypothesize that this decrease in resistance is due to the deformation’s tendency to strengthen the connection between these channels, providing a path of lower impedance to ground regardless of which sensor is being sampled. Though not modeled by the simulation, changes in the response sign should not affect the performance of the network if the response is still a function of the length of the sensor fibers under deformation. The usage of information theory metrics is hence vital here, as it is infeasible to analytically model the response of these sensor networks. Additionally, this strengthening effect is expected to depend not only on the location and depth of the probing, but also the direction in which the probe moves. In this study, we consider only vertical deformations, but the robot’s additional degrees of freedom could be used in future work to incorporate directionality into the training data. Figure 6’s narrow error bars indicate the repeatability of both morphologies’ resistive responses. One time series of five probes is plotted (purple) in Figure 6: Five decreases in resistance are clearly visible. The responses are significantly larger than any background noise in the system and, as such, simple signal filtering can later be applied to convert the response into a representative square wave. Over the five probes, the un-probed baseline resistance shifts by 50.1 Ω-23.5% of the average response magnitude. Over longer pressing periods, the baseline is found to `settle’ rather than maintain these high drift rates, which we hypothesize is due to the mechanical motion of connections during the early presses. Small shifts can still cause sudden jumps in baseline values during long-term implementation. By calculating the response magnitude relative to the baseline values before/after each probe, the effect of these small drifts is eliminated from any neural networks to which the values are input. Similarly, by fitting a square wave to the signal using total variation denoising, any overshoots (here, overshoots of the 25 Ω quantization level are occasionally seen) are removed.

### 3.2. Capacitive Sensor Characterization

**Figure 7:**
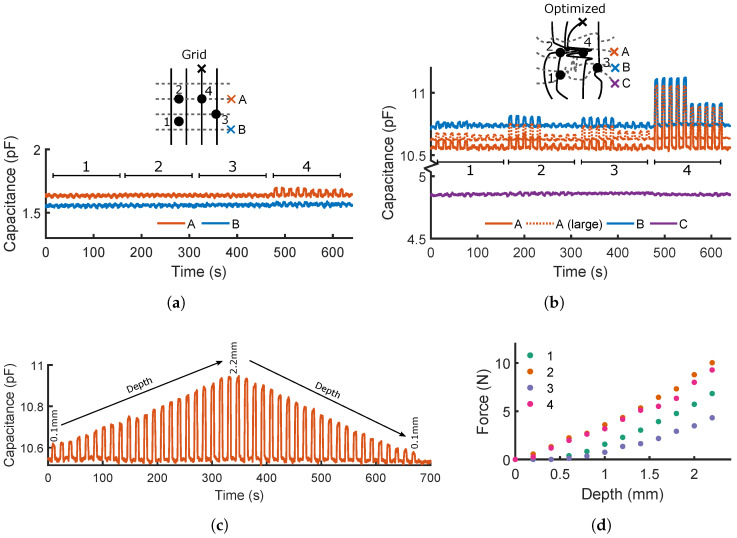
Capacitive responses of the two morphologies when probed and measured at different locations. (**a**) The grid morphology only yields a clear response when the intersection of the measured lines is directly probed at location 4. (**b**) The optimized morphology clearly responds at all 4 locations, discerning between probed depths of 1 mm and 2 mm. (**c**) Location 4 of the optimized morphology is probed at 22 different depths. (**d**) The compressive forces corresponding to each of the 22 depths at the 4 locations marked in Figure 7b.

The capacitances of the two networks are measured between pairs of sensors on opposite sides of the prints, marked by crosses in Figure 7a,b. One probe remains fixed at the black cross, whilst the other is moved between the colored crosses. For both morphologies, four locations are probed five times at depths of 2 mm then 1 mm, with Figure 7a,b showing the responses with time. Figure 7b also plots the measured responses at optimized location A when a larger probe with a diameter of 5 mm is fitted.

Given the thickness of the dielectric medium (0.4 mm) and the small surface areas between sensors, the limited magnitudes of the responses (∼$10^{-13}F$) are unsurprising: 2 orders of magnitude lower than the baseline capacitances of the sensor/logging setup, and comparable to the baseline drift in location B. Despite this, clear patterns arise from the four probed locations: particularly, the optimized morphology consistently returns responses of higher magnitude due to the increased surface area between the sensors acting as plates, compared to the small surface area (limited by the printed line widths) afforded between any two sensors in the square grid. At position 4, this intersection is exactly probed, and the only response discernible from background noise is returned. Conversely, the irregular morphology of the optimized sensor allows it to respond over a wider area; its responses are greatest when the distance between probe and the tracked sensors is minimized, suggesting that, by combining a number of capacitive responses, the probed locations could be inferred. The response of channel C, which encounters the least capacitive `plate’ area, shows by far the lowest response, at a level which is significantly affected by noise and baseline drift. A’s results are hardly affected by an increase in probe diameter (dotted orange line), despite a small shift in baseline capacitance between the two measurements. Future work will serve to better characterize the limitations of a fixed probe diameter on the transferability of the sensing capabilities, for both resistive and capacitive sensing.

The 1 mm-deep presses are always of lower magnitude than those with 2 mm, suggesting that the capacitive effect could be used to measure the depth of probing—if the corresponding material properties are known, then this allows the applied force to be inferred. To demonstrate this, Figure 7c plots optimized pair A’s response to 22 depths, ascending and descending between 0.1 and 2.2 mm. Higher responses are returned at greater depths, and the descending values closely match those measured during the ascension phase. If the resistive responses can be shown sufficient to predict the location of probing, capacitive sensing can provide further data to infer the depth of probing in this way. Figure 7d illustrates the near-linear correlation between depth of probing and applied force at the four locations (Section 5). Zero depth was taken to be the highest point at which any of the four locations started responding—in this case, locations 2 and 4 start responding simultaneously and behave very similarly. Locations 1 and 3 are further from the densely printed region, and are subsequently closer to the substrate and show a non-zero intercept. From this point, they behave with a similar gradient to 2 and 4, suggesting that if the surface height is known or controlled during printing, then all sensorized areas would respond similarly. If this mapping is known, then predicted depths are easily converted to applied force values.

These results act as a proof of concept of the prints’ abilities to perform as both resistive and capacitive sensors for localization and depth—combining the two types of measurement would provide further redundancy and robustness in the calculated probe locations and depths by increasing the joint entropy, and could be simultaneously measured from the gain and phase shift of applied AC signals. Additionally, the capacitive response could be used to measure the magnitude of an applied force (Figure 7c,d) along with its contact location, measured using the resistive response. Further developments of this optimization and fabrication method would seek to increase the effective signal-to-noise ratio by accounting for surface area in the objective function, and by minimizing the dielectric thickness. Due to its stronger response, all subsequent experimentation and results in this work focus on the resistive responses of the two morphologies, whilst bearing in mind that additional capacitive data could be used to improve these results and provide additional predictions of the applied forces.

### 3.3. Undamaged Sensors

To compare the behaviors of the printed morphologies with their simulated responses, each is probed at 5000 random locations, and the eight filtered sensor responses (See Section 5.3) are used to train an eight-input neural network to predict the location of probing (Section 5.3). Over the 5000 presses, the printed interface between the sensor channels and insulating substrate did not fail, suggesting the robustness of this fabrication method. However, the adhesive-bonded interface between the two substrates showed signs of delamination at its edges during the long-term experimentation, and fully-printed/different adhesion methods will be explored in future iterations.

**Figure 8:**
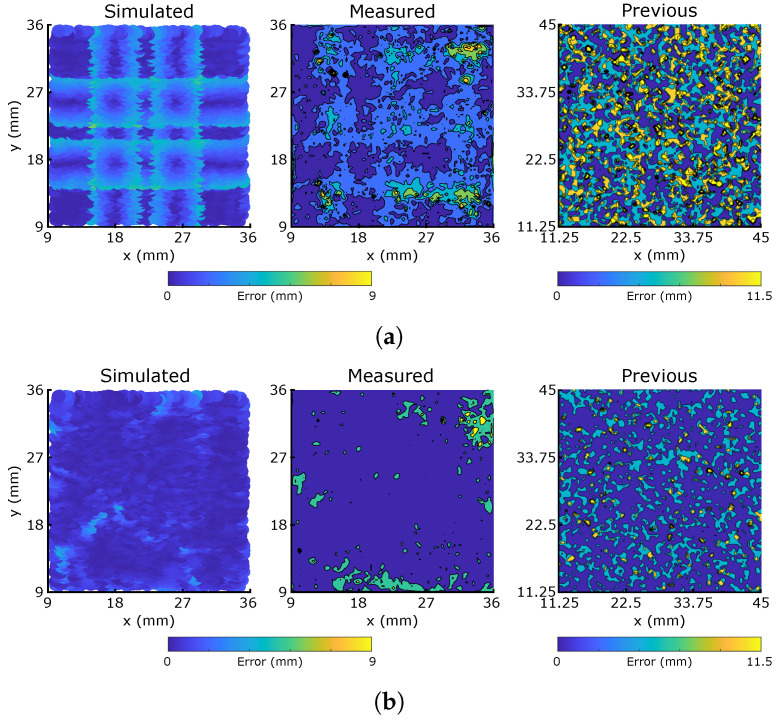
Simulated and measured x−y localization error distributions of single layer networks trained on 5000 presses of the two sensor morphologies, over the 25 × 25 mm area marked in Figure 5. The color bar covers the 9 mm grid size used for the physical prints. ’Previous’ refers to the manually fabricated sensor networks in [30], with an equivalently scaled color bar for the 11.25 mm grid size. (**a**) Grid; (**b**) Optimized.

The subsequent errors in predicted location over all 5000 samples are plotted in Figure 8, which shows the sim-to-real behaviors of the two morphologies (taken here to mean the differences in predicted error distribution). Both morphologies demonstrate similar macroscopic behaviors to those of the simulations, indicating that the simulator’s assumption that resistive dependence on local strain deformations is the effect most dominant in the sensor response. In the uniform morphology, the effects of the grid are apparent, yielding areas of minimum error at the centre of the bounded squares where there is most redundancy between the 8 responses, but higher errors around each sensor where, as simulated, the grid’s symmetry causes issues with localization. Conversely, there is no clear representation of the sensor morphology in the response of the optimized morphology, with errors more uniformly distributed across the characterization area. Small clusters of higher error emerge at the edges of the area, particularly near the top right corner, where nearby sensors are relatively sparse. The first column of Table 1 indicates the mean and median error magnitude for both sensors. At ∼2.5 mm, all of these values are remarkably small: less than one third of the 9 mm grid size, and less than one half of the 5 mm probe diameter. This demonstrates excellent performance of the material choice and fabrication method in producing unique and repeatable resistive responses.

Figure 8 also plots the equivalent heatmaps from the manually fabricated morphologies in [30], scaling the color bar via the grid size to account for size differences. Both printed morphologies match the simulated errors much better than those manually fabricated, with clearly lower average errors lessening the sim-to-real difficulties faced by the manual process. As identified in this previous work, the manual fabrication technique is labor-intensive and regularly introduces un-modelled pre-strain into the sensor fibers, causing damages and a large reality gap. Both of these effects are removed by the additive manufacturing and material technologies tested here, leading to a better match between the simulated and measured results. In addition, the significant decrease in thickness which printing facilitates a closer correlation with the 2D model, when compared to the 10 mm thickness required by the manual process. It should be noted that the previous work used different sensor and matrix materials as well as a comparatively larger-diameter probe, which are effects currently unaccounted for in the modelling and mapping stages of the resistive responses. A summary of key differences between the two experiments is presented in Supplementary Table S1. Though the higher number of samples in [30] might be expected to improve the previous training, other effects such as the smaller size of the hidden layer could introduce errors, and these comparisons should be viewed cautiously.

The optimized morphology network has a lower median error after training, reflecting its large consistently low-error areas containing only small regions of higher error. The grid has a lower mean error, though this is less uniformly distributed over the characterization area. By simultaneously measuring the capacitive responses to introduce more redundancy, we would expect to eliminate the optimized morphology’s higher errors and produce a fully uniform response, whilst the uncertainty in direction around the symmetrical grid lines is more difficult to remove.

### 3.4. Damaged Sensors

**Figure 9:**
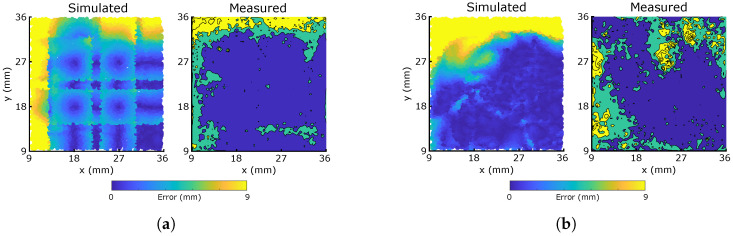
Simulated and measured x−y localization error distributions for the damaged sensor morphologies, before retraining of the neural networks. Two sensors are modeled as damaged (see Figure 5) by replacing their returned values with zeroes. (**a**) Grid; (**b**) Optimized.

To evaluate the printed sensors’ robustness to damage, we first examine a particular case in which one sensor from each side (marked red in Figure 3) is broken and returns no response. Considering a sensory skin deployed in a soft robotic application, there are two damaged sensor scenarios to be considered: in the first, the controller is unaware of the damage, and continues to infer tactile predictions under the assumption that both sensors are still operational. In the second, the controller has detected the damage, and is able to recalibrate its response accordingly. For the first case, we examine the subsequent errors using the trained networks of Section 3.3, simulating the damaged sensors by replacing their corresponding inputs in the data set with zeros during testing. The resulting error distributions, using the same scale as Figure 8, are given in Figure 9, with Table 1’s second column containing the mean and median error values. Despite a decrease in accuracy, all measured mean and median values remain impressively low, below the probe diameter. The optimized morphology has similar performance to the grid network for this sensor combination, though large regions of very low value errors are still prevalent throughout both distributions. The grid morphology’s main features match well between simulation and measurement, with the highest error regions occurring directly around the damaged sensors. The region of error in the lower right has a similar magnitude to the error in Figure 8, and may have arisen from an underperforming or loosely connected sensor during testing.

Though the optimized morphology’s error regions do not clearly align with those of Figure 9’s simulations, the measured errors are mostly *lower* than those predicted, suggesting that the complex interplay and non-uniformity between the multiple channels produces a series of unique responses which were not modeled by the simulator’s simple strain assumptions. Our ability to quickly 3D print and test new sensor morphologies allows these difficult-to-simulate advantageous effects to be exploited through physical optimization, an approach that is infeasible when the complex networks must be fabricated by hand. It is impractical to physically optimize a high-dimensional parametric model of a sensory system. Open parameters have to be narrowed down before physical optimization. Here, simulation models are vital.

**Figure 10:**
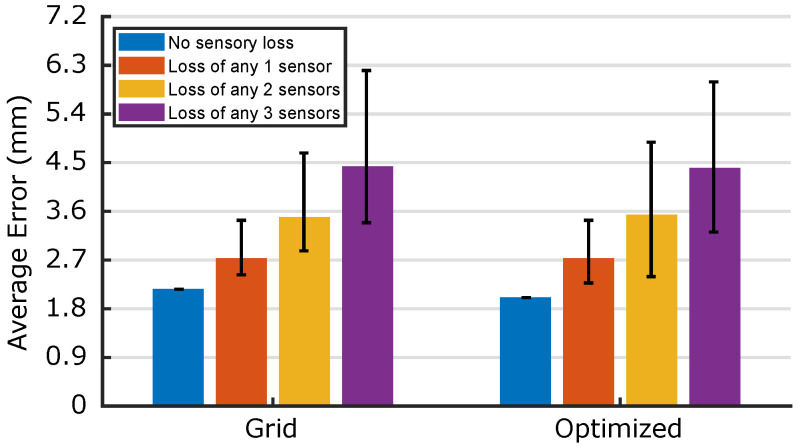
Robustness of the two networks experiencing the damage to any combination of ≤3 sensors without retraining. There are 8n possible combinations of losing *n* sensors: here, cases n=0,1,2,3 are considered. For all combinations, the median localization error is calculated, and its range plotted here.

To compare the robustness of the two morphologies, Figure 10 plots the average median error over all possible combinations of ≤3 damaged sensors: for *n* damaged sensors, 8n combinations are considered. In each case, the median localization error of the pretrained network is calculated, with the range of all 8n calculations represented by a single bar in Figure 10. The two morphologies behave very similarly: the optimized morphology outperforms the grid for the *n* = 0 and *n* = 3 cases, whilst the grid averages a marginally better response when *n* = 1 and *n* = 2. Though this suggests that there is little reason to prefer one morphology based on these robustnesses, it is noted that the optimized error bar minima are always lower than those of the grid, a result which extends when retraining is performed for all {n∈N|n<8}, i.e., for a given *n*, the error distribution with the lowest median error is always produced by the optimized morphology. For applications in which a particular sensor or combination of sensors is more likely to be damaged (such as those clustered near the leading edge of a locomotive robot’s soft skin), this suggests that morphologies can be developed with lower error increase than a 4×4 grid when these sensor/s are damaged. This knowledge can be used to aid the development of sensors with vulnerable or high-risk regions.

**Figure 11:**
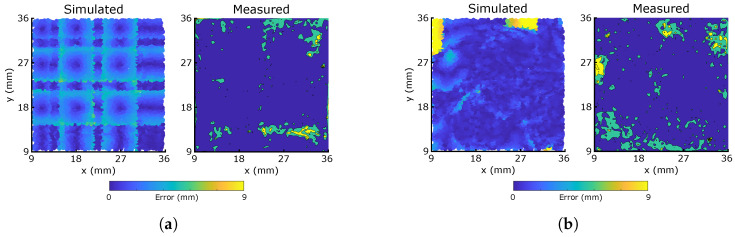
Simulated and measured error distributions for the damaged sensor morphologies, after retraining of the neural networks. The two damaged sensors (see Figure 5) are ignored, and the networks are trained from scratch with only six inputs. (**a**) Grid; (**b**) Optimized.

The second damage scenario, in which the controller knows to neglect the damaged sensors, is presented in Figure 11. To produce this, Section 3.3’s neural networks are restructured and retrained with only 6 inputs from the same data set: any responses of the two sensors marked in Figure 3a are ignored. In both cases, the controller is able to correct Figure 9’s large error regions to produce large regions of low error: in many areas, the measured responses perform *better* than the simulation, indicated by darker blue regions.

**Figure 12:**
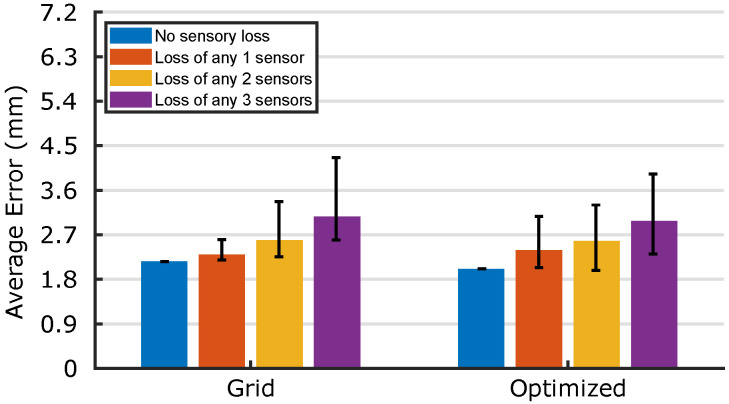
Robustness of the two networks experiencing the damage to any combination of ≤3 sensors after retraining. There are 8n possible combinations of losing *n* sensors: here, cases n=0,1,2,3 are considered. For all combinations, a new network is trained to ignore the damaged sensors, the median localization error is calculated, and its range plotted here.

As predicted by the simulation, the effect of errors is more localized and less symmetric in the optimized morphology. With 25% of sensors damaged, Table 1 indicates that both median errors increase by less than 0.5 mm from the undamaged case, demonstrating the excellent sensory redundancy which Section 2’s fabrication method is capable of providing. To truly compare the networks’ retraining capabilities, Figure 12 compares the median errors after retraining for any combination of up to three damaged sensors. Again, whilst neither morphology stands out as the best, the optimized morphology’s minima are less than those of the grid for {n∈N|n<8}. Additionally, the average errors of all damaged sensor cases have decreased from Figure 10 after retraining; the highest average error reported in Figure 12—4.26 mm—is smaller than the diameter of the probe, demonstrating a high retainment of the sensors’ locational accuracy even in the case of 37.5% total sensory damage. By coupling the trained networks with the sensors’ capacitive responses, and by introducing mechanisms for self-healing and damage detection [35], these quick-to-fabricate sensory skins pave the way towards the custom manufacture of truly universal soft sensory skins for wearables and soft robotics.

## 4. Conclusions

The ability to easily fabricate complex resistive sensor morphologies through additive manufacturing enables us to optimize and tune the properties of sensory skins to particular applications, including areas of uniformly low error or particularly high redundancy. With measured error distributions demonstrating consistently similar patterns to those predicted by simulations, we have demonstrated the approach’s potential to minimize the previously large reality gap faced when optimizing such morphologies.

The fabricated sensors can produce clean and repeatable tactile responses during measurements of resistance and capacitance, with minimal drift and overshoot. The combination of both sensitivity types shows promise in the design and production of sensors which are robust to significant damage, increasing redundancy with no corresponding increase in sensor complexity.

The governing strain-dependent resistive response mechanism observed in the measured sensors is the same as that assumed by the simple simulator, with a number of additional effects observed to emerge from crosstalk and from the sensor channel width. Though not included in the model, the non-uniform responses that these produce are advantageous to the neural networks, allowing them to accurately infer the probed locations and to often outperform the predicted results. The crosstalk between the sensors and the non-linear responses could explain why the optimized sensor and grid sensor perform similarly in their robustness to damage. The uniqueness of these sensory signals to particular areas of the optimized morphology provides a promising route for further development of multi-touch flexible sensory skins. Furthermore, the simplicity of the digital fabrication enables a quick method of testing these real-world effects for inclusion in the optimization process, creating custom sensor morphologies that match the desired behaviors.

## 5. Methods

### 5.1. Design of the Sensor Morphologies

In this section, we briefly describe the modeling and design approach for optimized morphology. A detailed overview can be found in [30]. A purely geometric model of the sensory skin is used for optimizing the sensory skin. Here, we assume that the sensor response is just a function of the sensor geometry and the shape of the contact surface. Each sensor morphology is parameterized with *N* variables. The number of sensors in a grid is denoted by 2M, where *M* is the number of sensors on each side. Each parameter corresponds to a point (with coordinates x,y) in space. Piecewise cubic hermite interpolation is used to derive the shape of the sensor from these *N* 2D coordinates. Two such sensory skins (M=4), optimized in the authors’ previous work [30], are shown in Figure 3, where the first square grid network is the most commonly used morphology for contact localization. Such morphologies are not ideal as for each location on the skin only two sensors will be active at a time, making it inefficient and more sensitive to damage.

Once the deformation shape and location of contact is known, the strain values in each sensor can be simulated with our geometric model. The model determines the strain values from the length of the sensor within the deformation area, scaled by the inverse of its distance from the center of the deformation. Once each morphology is parametrized, we can evaluate them using numerical IT metrics. We use joint entropy as a measure of robustness to loss of sensory data. For non-redundant localization tasks (where the sensor distribution is sparse with respect to the contact area), increasing the joint entropy also results in an average increase in localization accuracy. The joint entropy of the simulated parametric model can be estimated by a short, continuous and uniform sampling of the response across the sensor area. We use a genetic algorithm for finding the optimal morphologies. On top of optimizing the joint entropy of the sensory skin, we add additional penalties on the cumulative area of sensor crossing and the sharpness of the shapes to obtain the right trade-off between information content, learnability and ease-of-fabrication. For this work, we test an optimized shape from the authors’ previous work [30] that is designed to be robust to damages (Figure 5).

### 5.2. Characterization Methods

A Universal Robots UR5 robotic arm is used to characterize the sensor responses (Figure 5). A PLA probe-end effector is designed to provide a uniform pressure over a circle of diameter 5 mm: 56% of the 9 mm grid size. The centre of the probe can be sent to any point within a 25 mm square characterization area, shaded red in Figure 5, where it is used to depress the sensor’s surface by 2 mm. The UR5 can achieve these motions with a precision of 0.1 mm: approximately 5% of the lowest average error appearing in Figure 10 and Figure 12. Figure 5 figure also shows schematics and physical prints of the M=4 uniform grid and optimized morphologies which are compared throughout this work.

The underside of each printed grid is secured onto 2 mm-thick EVA foam using VHB tape, and secured firmly to the table. The compressibility of this foam enables the probe to provide substantial local deformations to the grid, reproducing responses similar to those during use as a flexible skin on the surface of a soft material.

To characterize the resistive responses, one end of each sensor is grounded and the other coupled to +5 V via a 1.2 kΩ resistor, creating a potential divider (Supplementary Figure S2). The voltages at the central nodes of the dividers are sampled using the analog input pins of an Arduino Pro Mini microcontroller, sent via serial connection to a PC, and recorded using Teraterm logging software. Conversion to resistance is performed after measurements are taken, such that the logged values are quantized into the 10-bit resolution of the microcontroller at 10 Hz.

The capacitive responses of each of the two morphologies are demonstrated by sampling the capacitance across the thickness of the insulating material. This is recorded using a Keysight E4980AL LCR meter at 10 kHz, which sends measurements to a host PC with a ∼30 Hz sampling rate. Figure 7d’s force values are recorded by securing the EVA foam onto the surface of a Kern 1200-2N balance, and increasing the depth of the UR5 probe in 1 mm increments.

### 5.3. Neural Network Implementation

Neural networks are used to predict the probed location on each sensor, given 8 inputs—one value from each sensor. To extract these values, the resistive responses of all 8 sensors are simultaneously recorded during the probing using the serially connected microcontroller. The time-series signals are filtered using total variation denoising (λ=10) [30,36], in order to convert all probed responses to a set of 8 representative values using the drift elimination approach discussed in Section 3.1. From these 8 inputs, the network aims to output a two dimensional x−y coordinate of the probe’s location. After splitting the 5000 data samples into 70:15:15% Training/Validation/Test sets, a separate 8-input → 100-neuron hidden layer → 2-output single layer neural network is trained using the Levenberg–Marquardt algorithm for each morphology. Unknown damages are simulated by replacing the channels under consideration with zeros and evaluating the error distribution using the pretrained networks. During the analysis of known damages to the sensory skins, the input layer size is adjusted to match the number of active sensors under consideration, and the network is retrained, i.e., ignoring 2 damaged sensors requires a 6-input network to be trained.

## Figures and Tables

**Table 1:** Mean (μ) and median (*M*) error values for the 6 plots in Figure 8, Figure 9 and 11.

	Undamaged	Damaged	Damaged + Retrained
$M_{A}\left(\mathrm{mm}\right)$	2.16	3.07	2.38
$M_{B}\left(\mathrm{mm}\right)$	2.01	3.53	2.46
$\mu_{A}\left(\mathrm{mm}\right)$	2.54	4.34	2.95
$\mu_{B}\left(\mathrm{mm}\right)$	2.65	4.82	3.28

## Supplementary Materials

The following supporting information can be downloaded at: https://www.mdpi.com/article/10.3390/mi13091540/s1, Figure S1: Flexibility and stretchability of a 0.3 mm printed sensor grid; Figure S2: A schematic of the resistive sensing setup, which consists of 8 potential dividers in parallel. The GND and 5V outputs and the analog inputs are all connected to a microcontroller. In this schematic the sensors are assumed separate, though some channels in the optimized print are in contact. This introduces additional resistances between the 8 branches, the effect of which can quickly be learned by the neural networks. Table S1: Flexibility and stretchability of a 0.3 mm printed sensor grid.

## Abbreviations

The following abbreviations are used in this manuscript:

IT	Information Theory
AC	Alternating Current
FDM	Fused Deposition modeling
PLA	Polylactic Acid
EVA	Ethylene-Vinyl Acetate
VHB	Very High Bond
