# Supplementary material for: 3D Printable Soft Sensory Fiber Networks for Robust and Complex Tactile Sensing
Source: Micromachines (Basel). 2022 Sep 17;13(9):1540. doi: 10.3390/mi13091540 (PMC9502117; doi:10.3390/mi13091540)
Supplement: Supplementary file 1 [file micromachines-13-01540-s001.zip › micromachines-1913880-supplementary.pdf]

Table S1. Key differences between the two works compared in Figure 8.

	**This Work**	**Thuruthel et al. [30]**
**Sensor material**	Styrene-based tri-block co-polymer & carbon black	Conductive thermoplastic elastomer (CTPE)
**Substrate material**	Styrene-based tri-block co-polymer	EcoFlex 00-20
**Fabrication method**	3D printing	Manual positioning & casting
**Thickness (mm)**	$0.3 \rightarrow 1.2$	10
**Grid size**	4×4	4×4
**Probed area (mm)**	27×27	33.75×33.75
**Probe diameter (mm)**	5	7.5
**Depth probed (mm)**	2	4
**Number of samples**	5000	7400
**Hidden layer size**	100	50

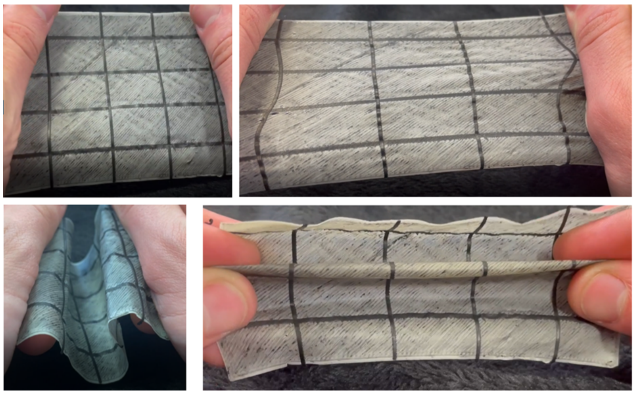

Figure S1. Flexibility and stretchability of a 0.3mm printed sensor grid.

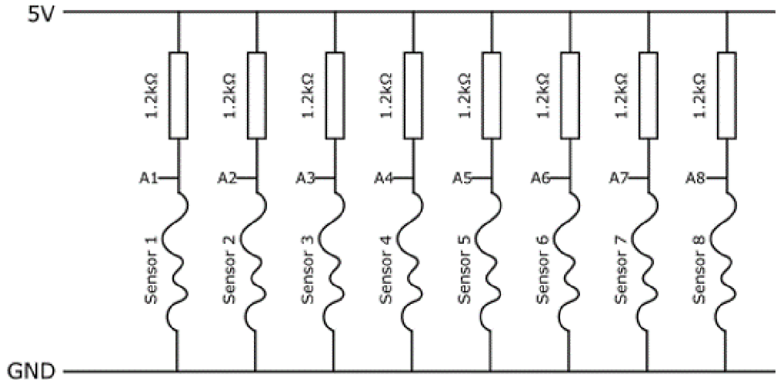

Figure S2. A schematic of the resistive sensing setup, which consists of 8 potential dividers in parallel. The GND & 5V outputs and the analog inputs are all connected to a microcontroller. In this schematic the sensors are assumed separate, though some channels in the optimized print are in contact. This introduces additional resistances between the 8 branches, the effect of which can quickly be learned by the neural networks.
